# Expedited Transition in the Wettability Response of Metal Meshes Structured by Femtosecond Laser Pulses for Oil-Water Separation

**DOI:** 10.3389/fchem.2020.00768

**Published:** 2020-09-29

**Authors:** Sharjeel Ahmed Khan, Vadim Ialyshev, Vyacheslav V. Kim, Mazhar Iqbal, Hamad Al Harmi, Ganjaboy S. Boltaev, Rashid A. Ganeev, Ali S. Alnaser

**Affiliations:** ^1^Department of Physics, American University of Sharjah, Sharjah, United Arab Emirates; ^2^Faculty of Physics, Voronezh State University, Voronezh, Russia

**Keywords:** superhydrophobic-superoleophilic, superhydrophilic underwater superoleophobic, vacuum aging, air aging, oil-water separation

## Abstract

Oil-water separation using super-wetting and the selective permeability of membranes for oil or water has great ecological and economic significance. We report on the transition of wettability response, from superhydrophilic underwater-superoleophobic to superhydrophobic-superoleophilic state, by nanostructuring stainless steel and copper meshes using ultrashort femtosecond laser pulses. Our approach is environment-friendly, chemical free, and efficient as it exploits the benefit of aging the processed samples in a high vacuum environment. We optimized the laser scanning parameters, mesh pore size, and aging conditions to produce membranes exhibiting an extraordinary separation efficiency of 98% for the oil-water mixture. A variation in the water and oil contact angles for different meshes is presented as a function of the laser scanning speed. Stainless steel meshes with 150 μm pore size and copper meshes with 100 μm pore size have demonstrated an excellent wettability response for oil and water phases. Vacuum aging causes rapid chemisorption of hydrocarbons on laser-structured surfaces in the absence of water molecules, rapidly transforming the wetting state from superhydrophilic to superhydrophobic.

## Introduction

Oil-water separation has great ecological and economic significance. Organic industrial waste mixed with water during metal processing, oil refining, and food processing requires efficient oil separation and treatment before disposal (Milić et al., 2013). Similarly, oil spill accidents are frequent due to the global surge in the demand for crude oil. Oil spills discharge millions of gallons of oil, which drastically affects marine life and the environment (Barron, 2012). Traditional oil separation techniques such as burning, skimming, and chemical dispersion, are partially effective, but they produce harmful byproducts that worsen the separation efforts (Barry et al., 2017). In recent years, the super-wetting behavior of 2D membranes or 3D adsorbent foams have been used because of their robust, low cost, environmentally friendly, and high separation efficiency characteristics (Gupta et al., 2017; Wang et al., 2018). Membranes with selective permeability toward oil and water phases can be achieved by inducing either superhydrophobic-superoleophilic or superhydrophilic underwater-superoleophobic wetting behaviors. Superhydrophobic-superoleophilic membranes are “water removing,” whereas superhydrophilic underwater-superoleophobic ones are “oil removing types;” and both have been targeted in strategies developed for addressing the oil-water separation problem.

Oil-water separation based on superhydrophobic-superoleophilic wetting behavior has been achieved with high separation efficiency by depositing SiO_2_ nanoparticles on filter papers and foams (Khan et al., 2017). Similarly TiO_2_ (Li J. et al., 2016; Bano et al., 2018) and ZnO (Feng et al., 2015) nanoparticles, with extreme wettability behavior, have been employed for oil-water separation. Moreover, techniques like sol-gel, electrodeposition, chemical etching, spray and dip coating of filter paper (Du et al., 2014), meshes (Zulfiqar et al., 2018), fabric (Sharma et al., 2019), and magnetic sponges (Wu et al., 2015; Beshkar et al., 2017) featuring super-wetting characteristics were employed to separate oil-water mixtures. Although these techniques can separate oil-water mixtures, they consume harmful complex chemicals, and they lack durability and longevity since they are prone to mechanical damaging of the coated layers, which deteriorates their performance. On the other hand, laser surface structuring has proven to be an effective and durable technique for controlling the wettability of superhydrophobic and superhydrophilic wetting states (Peethan et al., 2019; Boltaev et al., 2020). Femtosecond laser surface structuring has lately emerged as a robust, environmentally friendly, non-contact, and mask-less process that is capable of producing features with very fine resolution over large areas, which makes it suitable for a wide range of applications (Vorobyev and Guo, 2013).

Recently, laser-structured surfaces have been used for separating oil-water mixtures. Oil water separation by laser technique has been achieved either by drilling micro-through holes or by structuring metal meshes. Copper filters were created by drilling micron-sized holes in copper sheets followed by raster scanning to produce underwater-superoleophobic and underoil-superhydrophobic wetting states (Zhou et al., 2018). Additionally, copper filters fabricated by nanosecond lasers showing a superhydrophilic underwater-superoleophobic state were also reported to separate oil-water mixtures (Ha and Chu, 2016). Micro-through holes were also drilled in titanium (Ye et al., 2016) and aluminum (Li G. et al., 2016; Zhang et al., 2017) to produce superhydrophilic underwater-superoleophobic wetting states, whereas polytetrafluoroethylene (Yong et al., 2016) structured with femtosecond lasers led to a superhydrophobic-superoleophilic wetting state that has been employed for oil-water separation. Moreover, femtosecond laser-induced structuring of stainless steel (Yin et al., 2017) and titanium (Cao et al., 2019) meshes have been employed to produce superhydrophilic underwater superoleophobic membranes used for separating oil-water mixtures. Generally, femtosecond laser ablation is accompanied with functionalization steps to alter or retain the laser-induced surface wettability of materials (Liu et al., 2017; Zhou et al., 2019). In most cases, surface structures generated as a result of laser ablation demonstrate superhydrophilic underwater-superoleophobic properties depending on the exposure conditions and material properties, and in order to transform them to a superhydrophobic-superoleophilic wetting state, a low surface energy coating of complex chemical reagent is applied (Liu et al., 2017). Though some literature exists on the laser-tailoring of surface wettability and its application in oil-water separation, the use of femtosecond lasers to create efficient membranes is still in its nascent stage (Alnaser et al., 2019).

In this work, we report on the transition of wettability from superhydrophilic underwater-superoleophobic to superhydrophobic-superoleophilic states by nanostructuring stainless steel and copper meshes using a high repetition rate femtosecond fiber laser. The use of a high repetition rate fiber laser of superb beam profile enables the formation of precise structures on large areas of material surfaces in a very short time. We used different scanning speeds to form different structures of enhanced roughness on the meshes' surfaces. The structured meshes were aged for ~4 h in a high vacuum environment, and for 60 days in an ambient atmosphere. The results from aging in these two environments are compared.

## Experimental Arrangements

**Figure 1:**
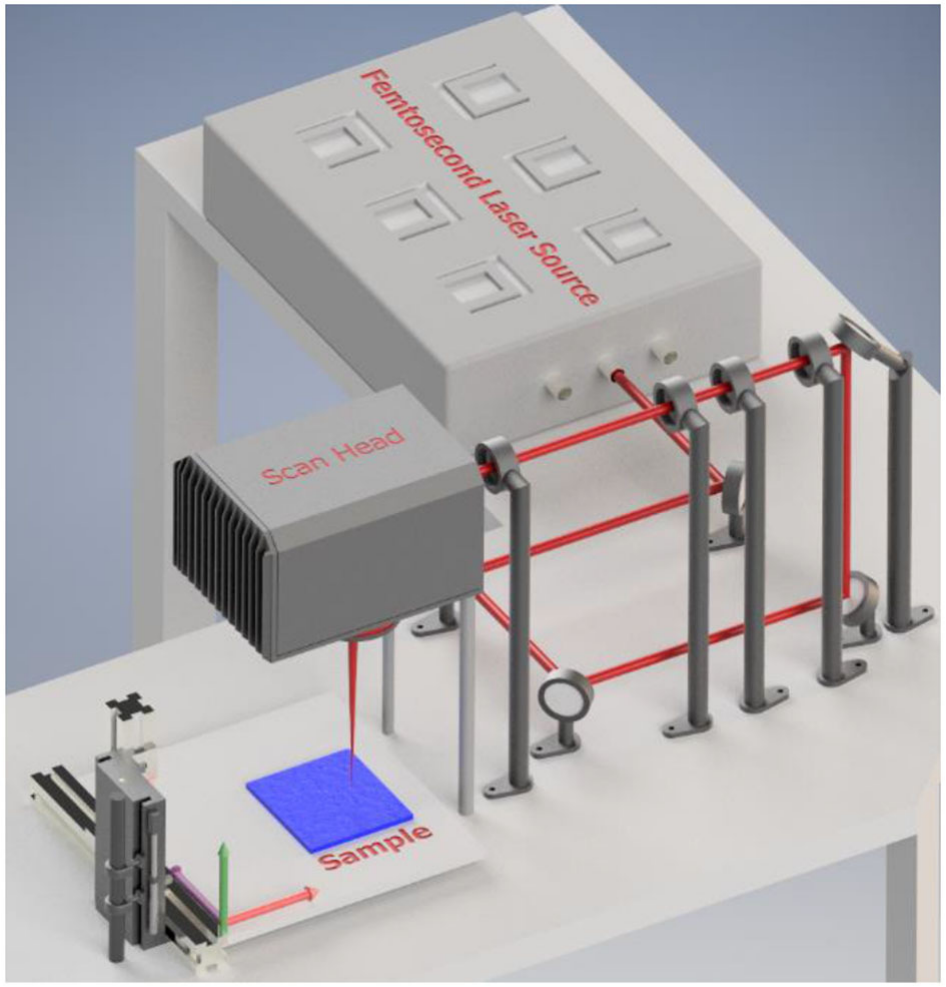
Experimental setup for the laser structuring of metal meshes with a high repetition rate femtosecond laser carried out at a scanning speed of (100–1200 mm/s) for stainless steel meshes and 100 and 300 mm/s for copper meshes. The laser beam was tightly focused on the meshes fixed on the 3D translation stage.

Stainless steel meshes (316 L) were used in the present study because of their high mechanical strength and commercial availability. Different meshes with various pore sizes and mesh wire thicknesses were purchased from Sigma-Aldrich (see Table 1). A high-power fiber-based laser amplifier system (AFS-UFFL-300-2000-1030-300 from Active Fiber Systems GmbH) with a central wavelength of 1,030 nm, repetition rate of 50 kHz, and a pulse duration of 36 fs was employed for nanostructuring of meshes. The laser polarization was linear with beam quality close to the diffraction limit (M^2^ < 1.3). The femtosecond laser surface structuring of the stainless meshes was carried out at different scanning speeds, ranging from 100 to 1200 mm/s, with a scanning width of 100 μm between the adjacent laser beam paths. Laser radiation with 5.0 W average power and 0.1 mJ pulse energy was focused on the stainless steel meshes by F-Theta lens and raster scanned using the scan head (FARO tech. Xtreme-20) as shown in Figure 1. The focused laser beam diameter was ~60 μm. Laser fluence of 3.5 J/cm^2^ per single pulse was used for surface structuring. Immediately after laser ablation, aging of the samples was performed either under high vacuum conditions (4.5 × 10^−5^ mbar) for 4 h or under normal atmospheric conditions for 60 days. The results from different aging methods are compared.

**Table 1:** List of the stainless steel and copper meshes, of different wire thickness and pore sizes, used in the femtosecond laser structuring.

**Mesh type**	**Wire thickness**	**Pore size**
SS-50#	200 μm	400 μm
SS-100#	100 μm	150 μm
SS-300#	30 μm	50 μm
SS-500#	25 μm	30 μm
Cu-100um	50 μm	100 μm
Cu-500um	250 μm	500 μm
Cu-800um	400 μm	800 μm

Additionally, copper meshes of 99.0% purity with different pore sizes purchased from Sigma-Aldrich were also ablated by femtosecond pulses with two different scanning speeds of 100 and 300 mm/s using the raster scanning method while keeping a distance of 100 μm between the two adjacent lines. The laser-structured SS and copper meshes were aged in air and vacuum and their wettability responses were compared.

Following the laser ablation, surface morphological analysis was performed using a scanning electron microscope (TESCAN VEGA3). For wettability characterization, water contact angle (WCA) and oil contact angle (OCA) were measured using Drop Shape Analyzer (KRUSS Germany) for the pristine surfaces of meshes, as well as for the laser-treated meshes aged in vacuum and in air. For the analysis of chemical moieties adsorbed on the laser structured surfaces after vacuum aging, FTIR spectra were recorded using ABB-MB 3000 series spectrometer attached to an attenuated total reflectance (ATR) accessory equipped with ZnSe crystal optics. Each curve represents 500 scans using the ATR cell as a reference.

For the oil-water separation tests, 15 ml of oil (n-hexane) and 15 ml of water (volume ratio of 1:1) were mixed together and then poured on the laser-structured mesh. To distinguish the two liquids we colored water with methylene blue. The flux (F) through the metal mesh used for oil-water separation was determined using F = V/ (A × *t*), where V is the volume of the liquid that permeates through the structured mesh of area “A,” and *t* is the time it takes to permeate through the mesh.

## Results and Discussion

### Stainless Steel (SS) Meshes

It is well-known that surface structures and microscopic surface roughness have the tendency to dramatically enhance the super-wetting state, and that only extreme wetting states will separate oil from water or water from oil. Any mediocre wetting state for oil and water will not separate these liquids with high efficiency. Therefore, the issue of partial wettability behavior should be addressed before the laser structured meshes are deployed in the separation of the oil-water mixture.

**Figure 2:**
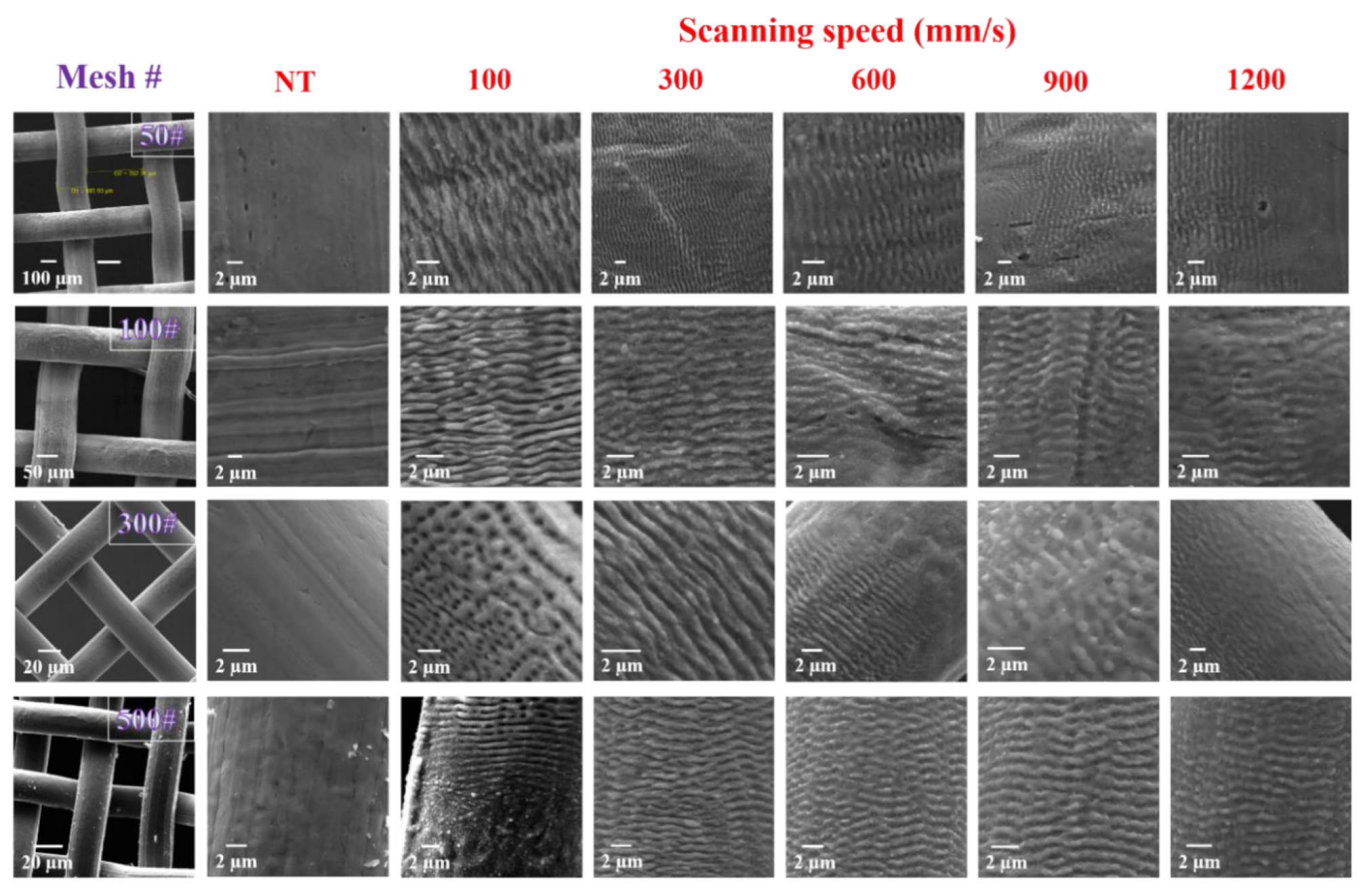
SEM of SS meshes structured at different scanning speeds (100–1200 mm/s). Laser induced periodic surface structures (LIPSS) were observed at different scanning speeds for various mesh sizes with a period of ~1 μm. In all those panels, the orientation of the LIPSS was always perpendicular to the laser polarization. At the two highest scanning speeds of 900 and 1200 mm/s non-treated regions on the meshes became prevalent and the depth of the periodic structures became significantly lower. Note, NT stands for non-treated meshes.

Laser-induced surface structures were created on the SS meshes of different mesh sizes at variable scanning speeds, ranging from 100 to 1200 mm/s. SS meshes (50#, 100#, 300#, and 500#) of different wire thicknesses and pore sizes were chosen for laser-induced structuring (left column of Figure 2). Mesh 50# has a wire diameter of 200 μm and pore sizes of 400 μm. Mesh 100# has a wire diameter of 100 μm and pore sizes of 150 μm. Mesh 300# has a wire diameter of 30 μm and pore sizes of 50 μm. Finally, mesh 500# has a wire thickness of 25 μm and pore sizes of 30 μm. Figure 2 shows the laser-induced periodic surface structures (LIPSS), of period ~1 μm, that were generated when irradiating the SS meshes at different scanning speeds. Changing the scanning speed of the focused laser beam will change the accumulated fluence on the surface, which results in forming different structures with different wettability responses. Depending upon the laser irradiation parameters, the treated surface can undergo the ablation process driven by either spallation or phase explosion mechanisms (Ivanov and Rethfeld, 2009). Spallation is defined as the expulsion of large liquid or solid particulates by the relaxation of laser induced stresses. Whereas phase explosion, also known as explosive boiling, involves the removal of materials due to explosive decomposition of superheated regions of the target surface (Zhigilei et al., 2009). At the slow scanning speed of 100 mm/s for SS meshes 300# and 500#, micro-pore formation and redeposition of micro-particles across LIPPS were observed. For scanning speeds of 300 and 600 mm/s, uniform LIPSS of period ~1 μm for all mesh types were formed. In comparison to the non-treated mesh surface, laser-treated surfaces were covered with LIPSS, which dramatically enhances their surface roughness. Supplementary Figure 1 shows the smoothness of the SS non-treated meshes compared to the laser-structured ones.

However, at the highest scanning speeds of 900 and 1200 mm/s, the depth of the LIPSS was reduced and random surface structures, in the form of protrusions, became prevalent.

**Figure 3:**
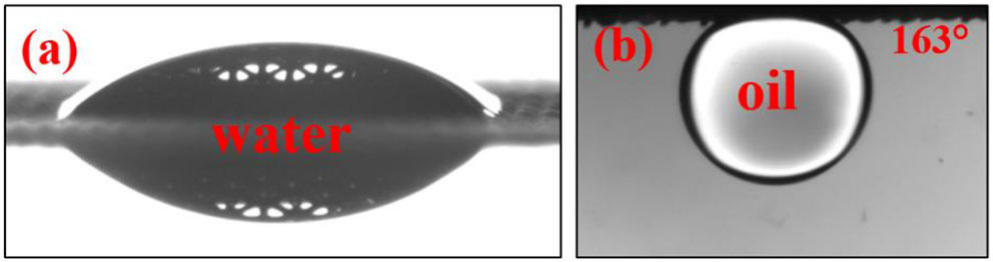
Wettability of femtosecond laser structured mesh demonstrating **(a)** superhydrophilic behavior in air and **(b)** underwater-superoleophobic characteristic.

We have measured the contact angle of the laser-structured surfaces immediately after ablation. The structured meshes demonstrated superhydrophilic response behavior immediately after ablation, as shown in Figure 3. This behavior is attributed to the formation of a metal oxides layer during laser structuring (Ngo and Chun, 2018). The water contact angle for the freshly ablated metal meshes was ~0° and the water droplet permeated through the mesh in ~0.1 ms, exhibiting superhydrophilic characteristics. Those superhydrophilic meshes showed a superoleophobic response under water, with oil contact angle (OCA) ranging from 155 to 163° across all scanning speeds and metal mesh types (see Supplementary Figures 2, 3 in the supplementary data file).

**Figure 4:**
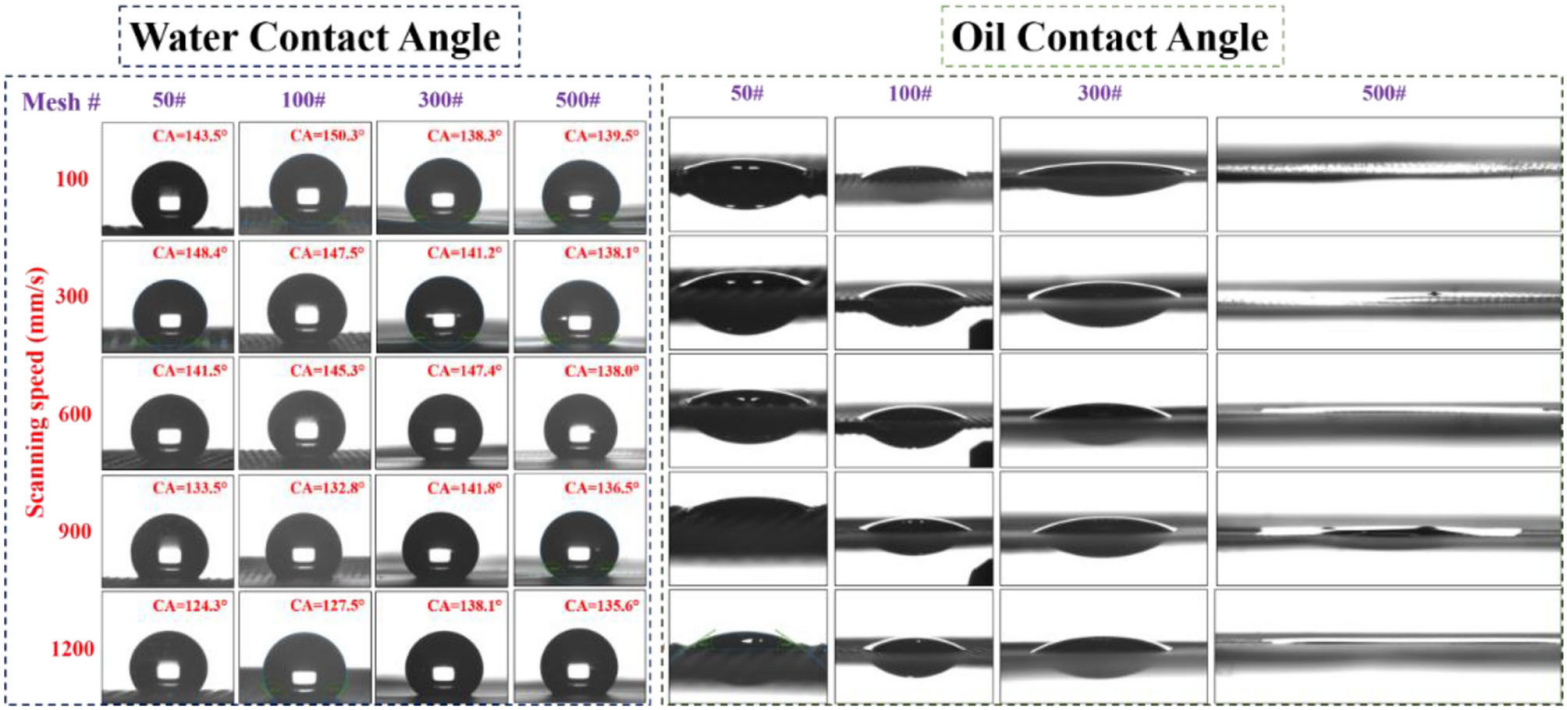
Water contact angle and oil contact angle measurements of SS meshes (50#, 100#, 300#, and 500#) stored in vacuum for 4 h after structuring with a femtosecond laser at scan speeds of 100–1200 mm/s.

**Figure 5:**
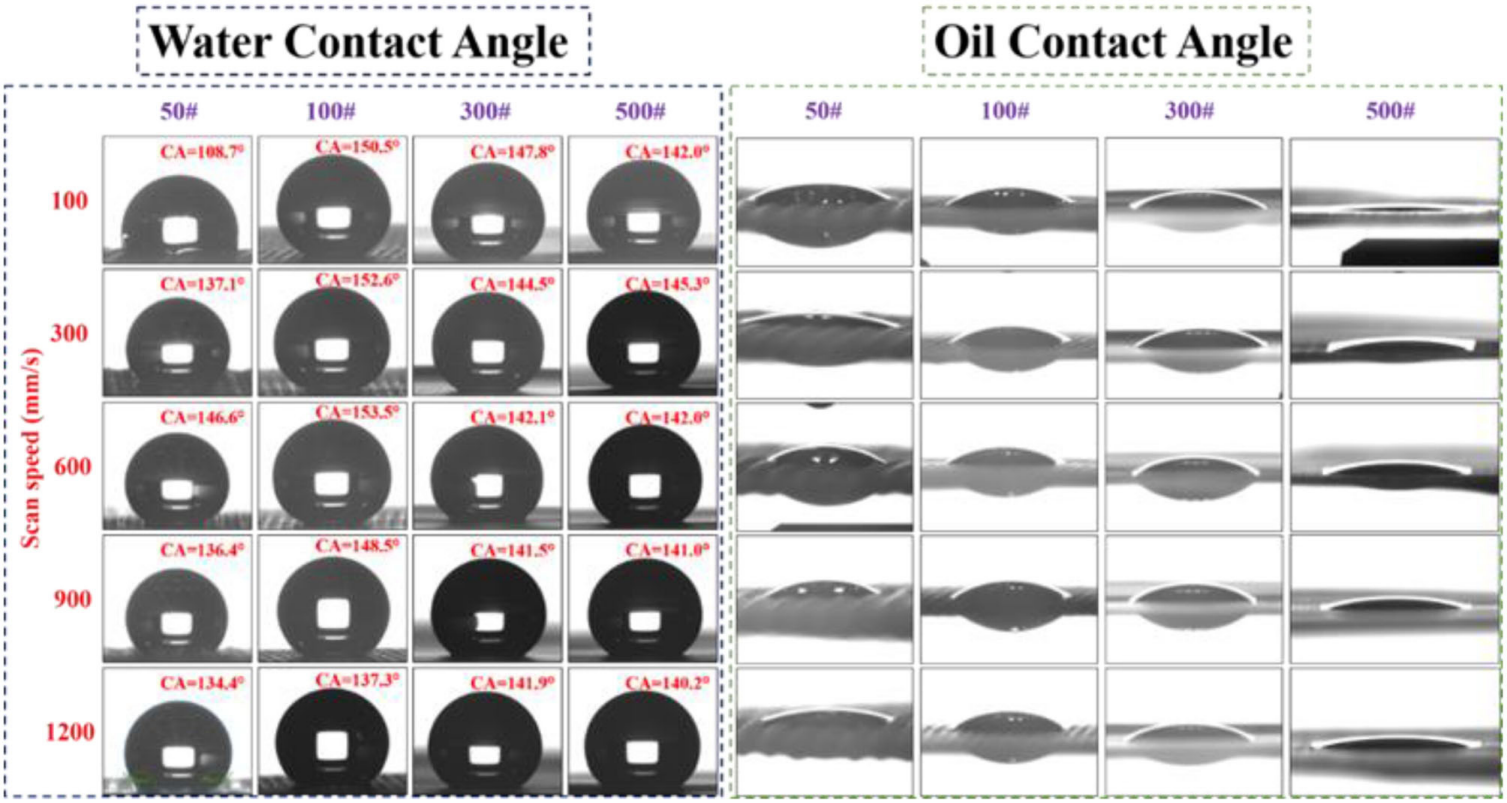
Water contact angle and oil contact angle of the SS meshes (50#, 100#, 300#, and 500#) ablated at different scanning speeds (100–1,200 mm/s) and measured after 60 days from laser surface structuring.

After long exposure in an ambient atmosphere for 60 days, or after 4 h of aging in vacuum, the superhydrophobic characteristic became dominant on the treated laser-structured meshes. Figures 4, 5 show the contact angle measurements of the laser-structured SS meshes at scanning speeds from 100 up to 1200 mm/s for different mesh sizes after vacuum and air aging, respectively. The water contact angle and the oil contact angle were determined by dosing 5 μl droplets of water and oil (n-hexane) on the laser treated areas. We observed significant variations in the wetting response of the samples aged in vacuum or air compared to the freshly (i.e., immediately after ablation) structured surfaces. For SS 50, 100, and 300# meshes, the oil seeped out, whereas for SS 500# mesh, the oil contact angle was close to 0° and oil did not seep out of the pores but rather spread uniformly due to capillary action in LIPSS for both vacuum-aged and air-aged meshes. Laser ablation followed by aging either in air or in a vacuum environment made laser structured surfaces more hydrophobic and oleophilic simultaneously. With the increase in the laser scanning speed, the permeability of oil slightly diminished, while still showing the oleophilic characteristics. The detailed analysis of water contact angle variations is addressed below.

**Figure 6:**
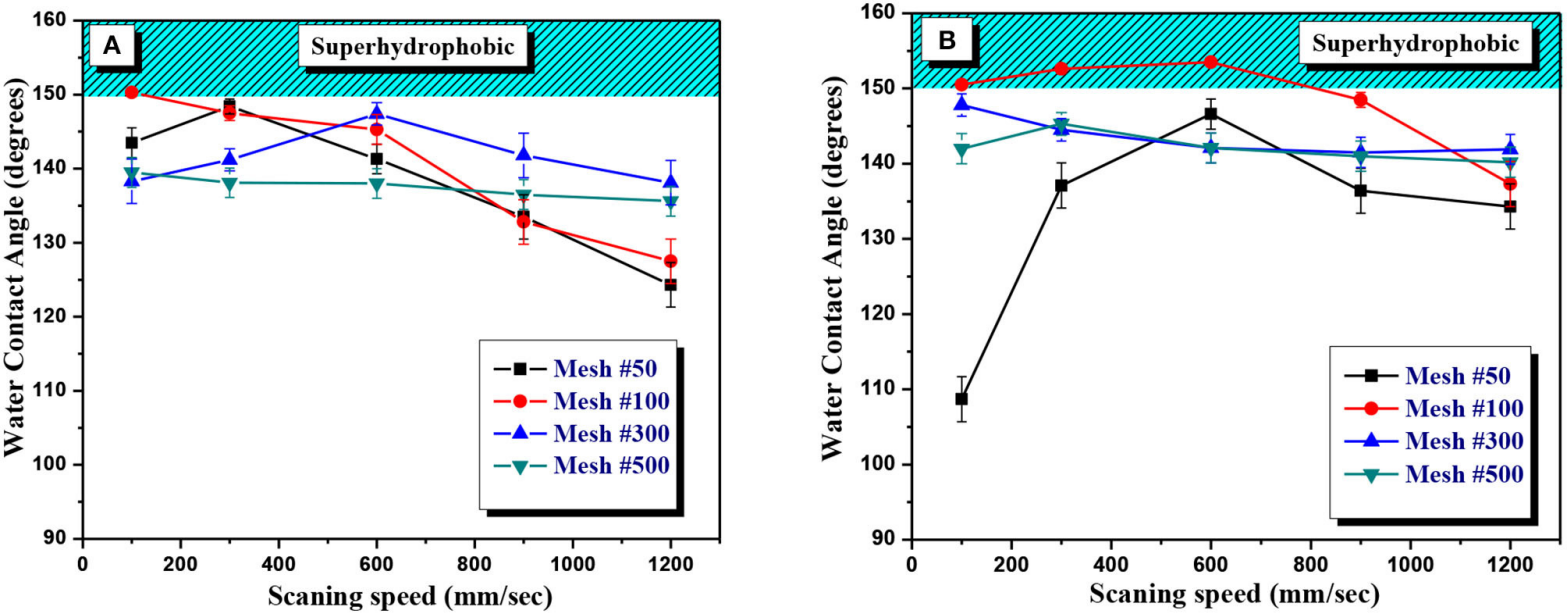
Water contact angle (WCA) of **(A)** vacuum-aged and **(B)** air-aged SS meshes (50#, 100#, 300#, and 500#) textured at variable scanning speeds by a femtosecond laser. Mesh 100# aged in air showed superhydrophobic characteristics at different scan speeds with a maximum contact angle of 153.5° at 600 mm/s scan speed.

Figures 6A,B shows the variations of the water contact angle at different scanning speeds in the case of SS meshes after vacuum and air aging, respectively. For SS mesh 50#, the water contact angle increased gradually up to 147° with the increase in scanning speed up to 600 mm/s and then decreased with further increases in scanning speed for vacuum-aged meshes. The air-aged samples also demonstrated an almost similar value for the contact angle at a scanning speed of 600 mm/s. However, at a slower scan speed (100 mm/s), the water contact angle was 108.7°, which is considerably lower than in the case of the vacuum-aged samples. The performance of air-aged SS mesh 100# showed the strongest superhydrophobic characteristics at a scanning speed of 600 mm/s with a contact angle of 153.5°. The random surface structures formed at this scanning speed largely increased the surface roughness, thus yielding a strong superhydrophobic response. For SS mesh 300#, the water contact angle of the vacuum-aged sample increased when increasing the scanning speed up to 600 mm/s and then decreased with an increase in the scanning speed. Whereas, for the air-aged mesh, the contact angle decreased from 147.8° to 141.9° with an increase in scanning speed. For SS 500# mesh, the water contact angle showed insignificant variation with the increase in scanning speed for the vacuum-aged samples, with an average contact angle of ~137°. However, for air-aged samples, the contact angle value was mostly close to 142°, with a maximum value of 145.3° at 300 mm/s scanning speed. Meanwhile, all meshes demonstrated a superoleophilic response for the n-hexane oil with the contact angle close to zero, allowing oil to seep through the mesh within milliseconds.

As discussed above, laser structured SS meshes that showed superhydrophilic underwater-superoleophobic behavior immediately after laser ablation had ceased to sustain their initial wetting state and they transformed toward the superhydrophobic-superoleophilic wetting state after aging in vacuum or aging in an ambient atmosphere. As shown in Figure 6, significantly faster (over only a few number of hours) variation in the wettability behavior of the laser structured meshes was observed after vacuum aging as opposed to aging for months in an ambient atmosphere (Figure 6A). Moreover, to test the sustainability of the superhydrophobic state on the meshes that were aged in vacuum for a few hours, we repeated the contact angle measurements after keeping those samples exposed to air for many weeks; yet they showed the same superhydrophobic behavior. Our observations on the transition in the wettability response, from superhydrophilic to superhydrophobic, under a high vacuum environment, of the structured metal surfaces are also consistent with previously reported data (Ngo and Chun, 2018). As has been reported earlier (Jagdheesh et al., 2017; Ngo and Chun, 2018), the wettability measurements of laser structured metal mesh immediately after laser ablation demonstrates superhydrophilic behavior due to the large presence of metal-oxides formed as a result of laser ablation. The unsaturated cations-anions formed after laser ablation stabilize themselves by heterolytic dissociative adsorption of H_2_O molecules from the atmosphere, giving birth to a hydroxylated layer over a metal-oxides layer (Jagdheesh et al., 2017). This hydroxylated layer has high affinity to adsorb water molecules through hydrogen bonding, which explains the hydrophilic nature of laser textured surfaces. However, it has been well established that the presence of –OH groups favors the chemisorption of non-polar hydrocarbons (short chained) from the surrounding environment. This chemisorption, which takes place via esterification, yields to chemisorbed carboxylates that cause an evolution into a hydrophobic state over weeks of aging in an ambient atmosphere. Moreover, superhydrophilic to superhydrophobic wetting transition can be expedited by storing the structured meshes under high vacuum. This rapid transition is due to the low partial pressure of H_2_O vapors under high vacuum compared to air atmosphere. The chemisorption of non-polar hydrocarbons takes place quickly on the laser-induced structures under high vacuum replacing the reactive hydroxylated site (see Figure 9 discussion). As the passive layer of water molecules on –OH groups is almost absent under vacuum, adsorption of hydrocarbons sourced from the oil in the pumps becomes faster (Hauschwitz et al., 2019). A vacuum of 4.5 × 10^−5^ mbar for 4 h suppresses the passivation of –OH sites with H_2_O molecules due to the absence of humidity in the chamber in comparison to the normal atmosphere. However, there were slight variations in the degree of superhydrophobicity between the structured samples stored in air and those stored in vacuum; the wetting transition by aging in vacuum was rapid and led to an averaged hydrophobic contact angle ~140°, which is significantly higher than the initial contact angle (~0°) of the freshly laser-ablated surfaces.

### Copper Meshes

**Figure 7:**
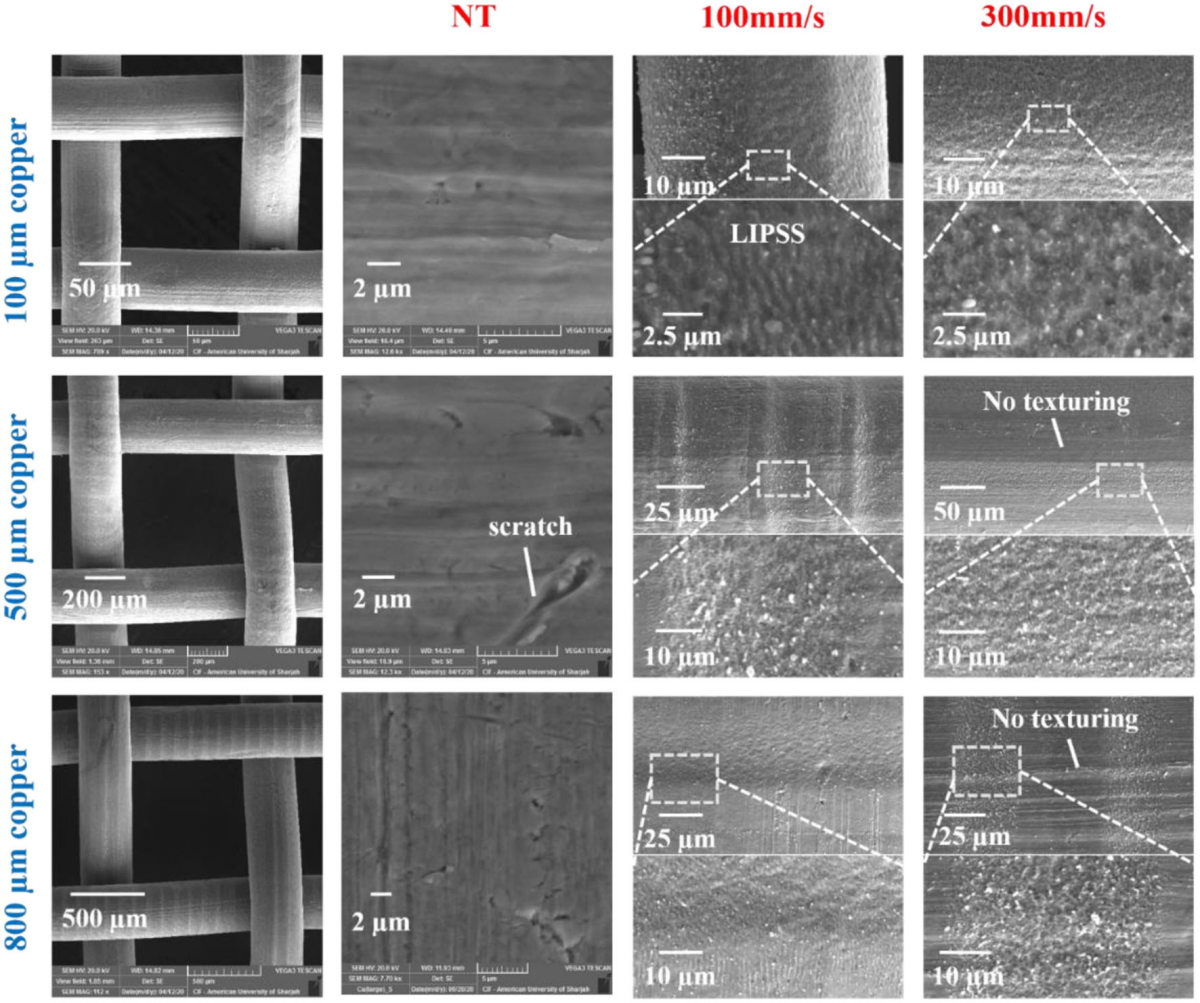
SEM images of three copper meshes (100, 500, and 800 μm pore sizes) structured by a femtosecond laser at scanning speeds of 100 and 300 mm/s. Laser treatment allowed the formation of LIPSS and rough surface structures. Some regions remained non-textured for meshes of 500 and 800 μm pore sizes at scanning speeds of 300 mm/s. Note: NT stands for non-treated mesh.

Copper meshes of 100, 500, and 800 μm pore sizes with wire diameters of 50, 250, and 400 μm, respectively (left column of Figure 7), were ablated with a femtosecond laser and then stored in air and vacuum environments. Figure 7 shows the SEM images of the structured copper meshes at two scanning speeds (100 and 300 mm/s). The formation of laser-induced structures on the copper meshes changed their surface color to grayish black. At an 100 mm/s scanning speed, LIPSS covered with micro- and nanoparticles were formed on the structured surface of the meshes; the average period between ripples was ~1 μm. For 500 and 800 μm copper meshes structured at a scanning speed of 300 mm/s, we found non-textured areas similar to those of pristine copper mesh. Moreover, at a 300 mm/s scanning speed, random nanostructures with protrusions were observed along the laser beam path.

**Figure 8:**
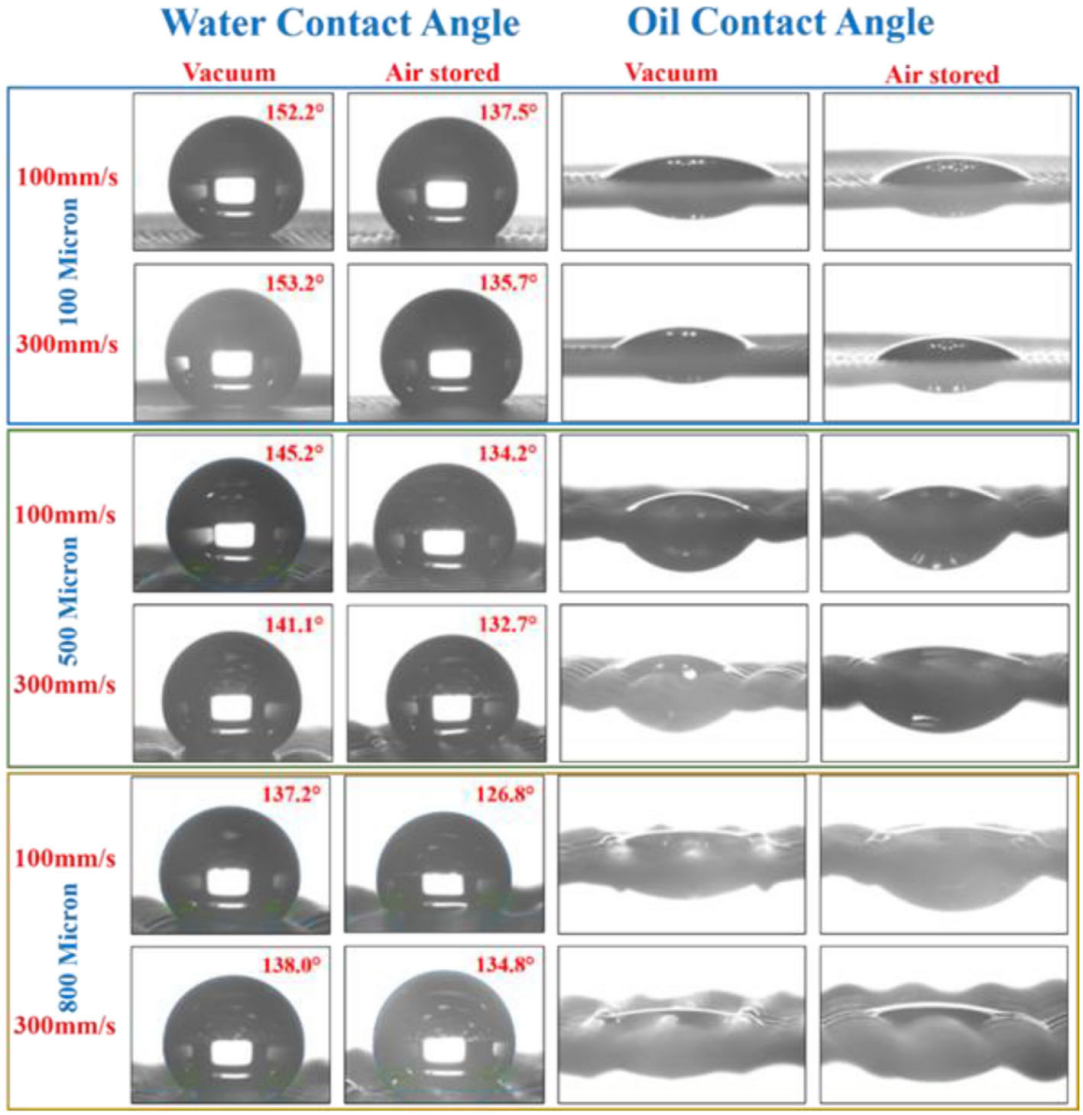
Water contact angle and oil contact angle measurements of the copper meshes of different pore sizes (100, 500, and 800 μm) after laser structuring at 100 and 300 mm/s scanning speeds followed by aging in a vacuum for 4 h and in air for 60 days.

Figure 8 shows the water and oil contact angle measurements of the laser structured copper meshes modified at two different scanning speeds followed by vacuum aging for 4 h and air aging for 60 days. The structured 100 μm copper mesh demonstrated a strong superhydrophobic response at 300 mm/s scanning speed after vacuum storage with a water contact angle of 153.2°. Notice that, under similar conditions, a water contact angle of 135.7° was obtained for air-aged samples. The same tendency of achieving a higher water contact angle after vacuum storage was observed for each copper mesh and at different scan speeds. Meanwhile, the oil drops permeate through the mesh, exhibiting superoleophilic response. The laser-processed 500 and 800 μm copper meshes also showed hydrophobic characteristics after vacuum and air aging. Water contact angles of 145.2° and 141.1° were obtained for 500 μm copper mesh kept in a high vacuum after laser structuring at scanning speeds of 100 and 300 mm/s, whereas samples aged in air showed smaller water contact angles (134.2° and 132.7°, respectively). A similar trend was observed for the 800 μm mesh with a water contact angle of 137.2° for vacuum-aged and 126.8° for air-aged samples structured at a 100 mm/s scanning speed. Meanwhile, the oil contact angle of the samples showed high permeability for oil exhibiting superoleophilic characteristics.

**Figure 9:**
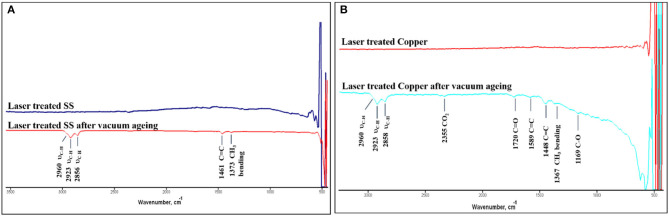
ATR-FTIR spectra of the Laser treated **(A)** SS and **(B)** Copper before and after vacuum aging.

Figure 9 shows the ATR-FTIR spectra for the laser structured stainless steel (Figure 9A) and copper (Figure 9B) metal surfaces before and after vacuum aging for samples prepared at scanning speeds of 100 mm/s. As shown in Figure 9, the laser treated samples lack the major IR modes in the regions 2,800–3,000 and 1,100–1,750 cm^−1^, which are associated with hydrocarbon attachments to the surface. This is expected since the laser treated samples were strongly hydrophilic when the contact was measured immediately after structuring. However, the surface hydroxyl and the water adsorbed signatures are weak in nature and hard to detect using the current ZnSe crystal. In contrast, the ATR spectra for the laser treated metal surfaces aged in vacuum showed IR modes associated with the adsorption of hydrocarbons on the treated surfaces. Specific bands appear at 2,960, 2,923, and 2,858 cm^−1^ assigned for C-H stretching modes for aliphatic -CH_3_ and -CH_2_- moieties. These bands are also associated with the υ_CH3_ bending mode that appears at 1,370 cm^−1^ in both vacuum-aged metals. Further signatures appear at 1,460 cm^−1^ assigned to the υ_C = C_ stretching modes. In addition, the vacuum-treated Copper sample showed additional bands at 1,720 and 1,169 cm^−1^ that were assigned for C=O and C-O-C stretching modes, respectively. In summary, the laser treated metal surfaces in the vacuum chamber facilitate the adsorption of organic hydrocarbons on the surface that leads to the aforementioned superhydrophobic behavior observed on the vacuum-aged mesh surfaces.

We showed that storing laser-processed meshes in high vacuum environments for a few hours is an efficient approach to fabricate, in a short time, superhydrophobic-superoleophilic meshes for oil-water separation applications. We also demonstrated the formation of wettability characteristics that surpass those reported previously using picosecond pulses, which also used air, vacuum, or carbon rich atmospheres as storing environments (Long et al., 2015). The maximal water contact angle, using picosecond laser pulses, was reported to be 120°, while in our case of the vacuum aging of the copper meshes fabricated by 36 fs pulses, the contact angle reached values up to 153.5°, thus demonstrating the superb superhydrophobicity behavior of our samples. The enhanced water contact angle in our study can be attributed to the formation of deeper periodic structures possessing sharper edges of the nanoripples created by femtosecond pulses. Such structures, demonstrating stronger local fields in the vicinity of sharp-edged nanoripples, have the tendency to better adsorb large amounts of hydrocarbons from the oily pollutants in the vacuum pumps or walls of the vacuum chamber than the structures formed by longer (picosecond) pulses.

**Figure 10:**
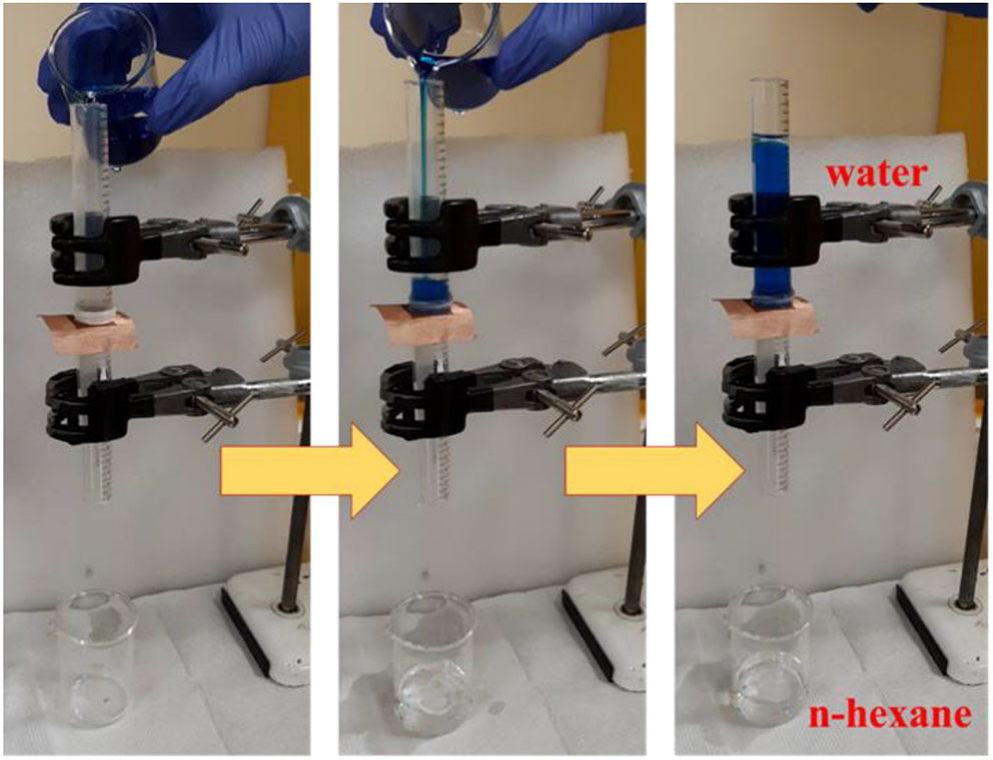
Demonstration of oil-water separation using laser structured and vacuum-aged copper mesh prepared at a scanning speed of 300 mm/s. The water is dyed with methylene blue for better identification of the two liquids. Oil passes through, while water retains above the mesh that possesses a superhydrophobic-superoleophilic characteristic.

The produced copper and SS meshes, which showed superhydrophobicity and superoleophilicity after vacuum and air aging, can be tested in the separation of oil-water mixtures into two different components by blocking water and allowing oil to permeate. Figure 10 shows the capability of the laser-structured copper mesh that was aged in vacuum for separating the oil-water mixture. The mesh was placed such that laser-treated surface faced the mixture. The oil-water mixture was poured on the laser structured mesh that features high permeability for n-hexane (oil). For identification, the water is dyed with methylene blue. When the oil-water mixture was poured on the laser structured surface of the copper mesh, the oil passed through the mesh and was collected in the beaker under the tube, while water was blocked in the tube above the mesh (right panel). The gravity driven separation occurred rapidly in no more than 15 seconds with a permeate flux (F) of 50 Lm^−2^h^−1^ and yielded an excellent performance of 98% separation efficiency, with little or no water in the separated oil (see also Supplementary Video 1).

## Conclusions

In conclusion, freshly prepared laser structured surfaces demonstrating superhydrophilic underwater-superoleophobic behavior were transformed to a superhydrophobic-superoleophilic wetting state after aging in air and in vacuum. We have demonstrated the rapid transition in wettability state of the freshly femtosecond laser-structured surfaces of SS and copper meshes, from superhydrophilic underwater-superoleophobic to superhydrophobic-superoleophilic using vacuum aging, and compared it with air aging. Copper meshes of 100 μm pore size processed by 36 fs pulses at scanning speeds of 300 mm/s, followed by vacuum aging, have shown excellent superhydrophobic-superoleophilic characteristics as the final stage of wettability. SS 100# mesh with a pore size of 150 μm, which was textured at a scanning speed of 600 mm/s and aged in air, demonstrated extreme wettability contrast for water and oil, respectively. The maximum water contact angle achieved was 153.5° and oil contact angle was 0°. Our systematic study on the thickness and pore size of the copper and SS meshes, the laser scanning speed, and aging environments provides guidance toward employing femtosecond laser-structured meshes in separating oil-water mixtures with high efficiency that can reach 98% and higher separation. The transition in wettability of femtosecond laser structured metal meshes from a superhydrophilic underwater-superoleophobic to superhydrophobic–superoleophilic response is achieved by a rapid, facile, environmentally friendly, chemical-free, and novel vacuum aging technique.

## Data Availability Statement

The raw data supporting the conclusions of this article will be made available by the authors, without undue reservation.

## Author Contributions

AA conceived the experiment. SK performed the laser structuring measurements. VK and MI helped with the laser operation and optimization. SK, VI, GB, and HA characterized the processed samples. AA, SK, and RG analyzed the results and wrote the manuscript. All authors discussed the results and contributed to the final manuscript.

## Conflict of Interest

The authors declare that the research was conducted in the absence of any commercial or financial relationships that could be construed as a potential conflict of interest.
