# Supplementary material for: Expedited Transition in the Wettability Response of Metal Meshes Structured by Femtosecond Laser Pulses for Oil-Water Separation
Source: Front Chem. 2020 Sep 29;8:768. doi: 10.3389/fchem.2020.00768 (PMC7550779; doi:10.3389/fchem.2020.00768)
Supplement: Supplementary file 2 [file Data_Sheet_1.docx]

**Expedited Transition in the Wettability Response of Metal Meshes Structured by Femtosecond Laser Pulses for Oil-Water Separation**

Sharjeel Ahmed Khan^1*^, Vadim lalyshev ^1^, Vyacheslav V. Kim^1^, Mazhar Iqbal^1^, Hamad Al Harmi^1^, Ganjaboy S. Boltaev^1^, Rashid A. Ganeev^1,2^, Ali S. Alnaser^1*^

*^1^ Department of Physics, American University of Sharjah, Sharjah 26666, UAE*

*^2^ Faculty of Physics, Voronezh State University, Voronezh 394006, Russia*


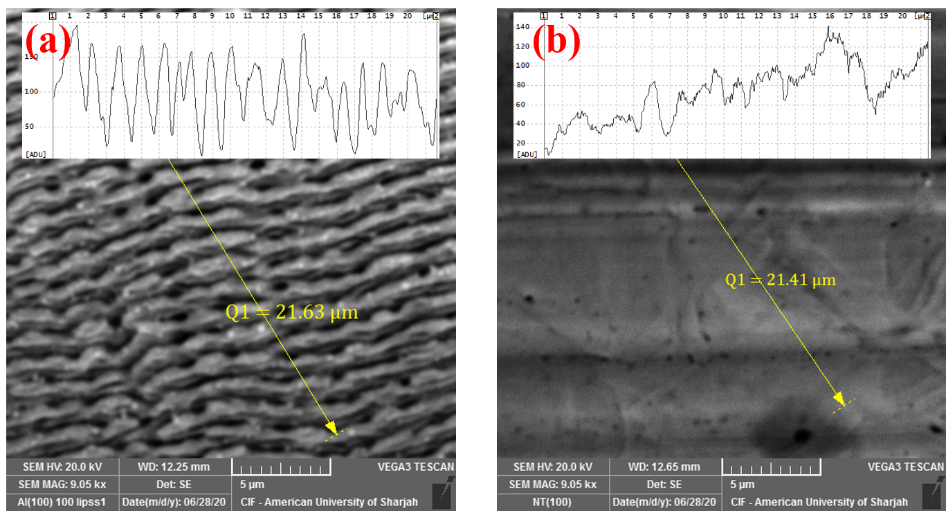


Fig S-1: SEM of (a) fs laser structured SS mesh at 100 mm/s scanning speed showing the formation of LIPSS. (b) SEM of non-treated (NT) mesh surface. Inset represent the roughness profile across the line.

Fig S-1 showing the surface profile of LIPSS formed on laser treated mesh for scanning speed of 100mm/s compared to the non-treated (NT) mesh. The surface roughness is much higher for the laser structured mesh as presented in the inset across the line on the SEM image Fig S-1(a) compared to NT mesh Fig S-1(b). It is evident that laser structuring generates highly rough surfaces.


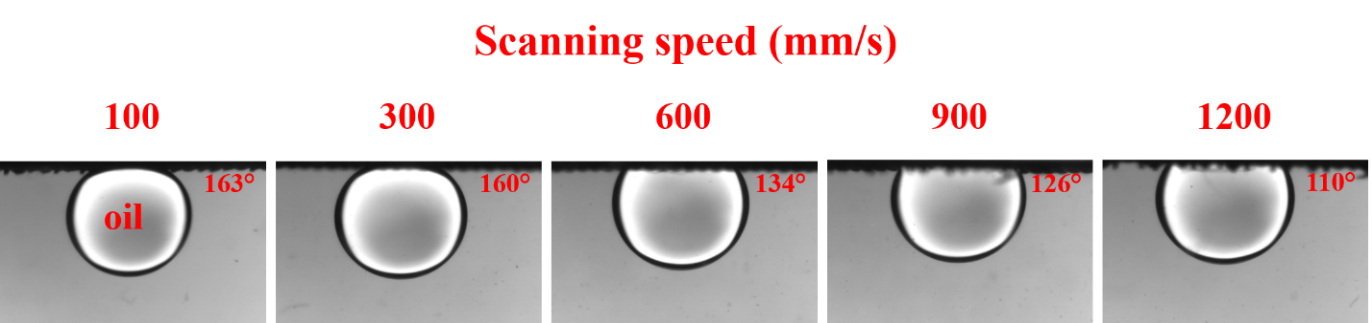


Fig. S-2: Underwater oil contact angle (OCA) by n-hexane oil for laser structured SS mesh 100# structured at different scanning speeds.


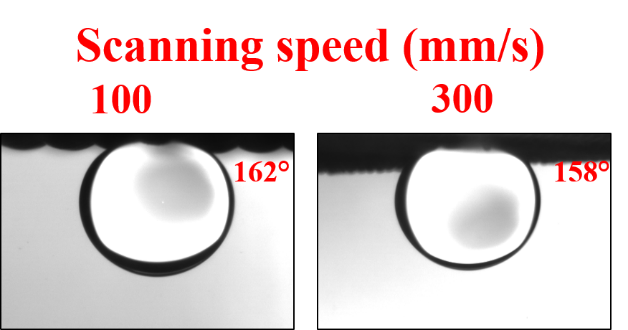


Fig S-3: Underwater oil contact angle (OCA) by n-hexane oil for laser structured Cu (100um) mesh processed at 100 mm/s and 300mm/s scanning speed.

Underwater oil contact angle (OCA) on the laser structured metal mesh was measured after 2 days of air ageing. Oil droplet (n-hexane) of ~8µl is placed on the surface of laser structured SS mesh 100# submerged in water (Fig S-2). The OCA for the 100 mm/s and 300mm/s is 163° and 160° respectively. With the increase in scan speed the OCA decrease rapidly. The decrease in OCA may be accounted to a decline in hydrophilicity by adsorbing air borne hydrocarbons. Similar underwater-OCA measurements were performed for copper mesh as shown in Fig S-3 for scanning speed of 100 and 300mm/s.
